# First prospective outcome data for the second-generation multigene test Endopredict in ER-positive/HER2-negative breast cancer

**DOI:** 10.1007/s00404-020-05771-4

**Published:** 2020-09-09

**Authors:** Johannes Ettl, Sophie-Isabelle Anders, Alexander Hapfelmeier, Stefan Paepke, Aurelia Noske, Wilko Weichert, Evelyn Klein, Marion Kiechle

**Affiliations:** 1 Department of Obstetrics and Gynecology, School of Medicine, Technical University of Munich, Klinikum rechts der Isar, Munich, Germany; 2 Institute of Medical Informatics, Statistics and Epidemiology, School of Medicine, Technical University of Munich, Klinikum rechts der Isar, Munich, Germany; 3 Institute of Pathology, School of Medicine, Technical University of Munich, Klinikum rechts der Isar, Munich, Germany

**Keywords:** Breast cancer, Prognostic biomarker, Predictive biomarker, Endopredict, Adjuvant chemotherapy, Endocrine therapy

## Abstract

**Purpose:**

Prospectively collected outcome data of patients (pts) whose adjuvant systemic therapy recommendation was based on the clinico-molecular test EndoPredict^®^ (EP) are presented.

**Methods:**

Pts with ER-positive, HER2-negative early breast cancer with 0–3 positive lymph nodes were enrolled. The EP was carried out on all tumor samples. Pts were evaluated for treatment compliance, local recurrence, distant metastases and overall survival. Censored time-to-event outcomes were analysed by Cox proportional hazards models. Additional estimates of the event-free-survival were calculated by the Kaplan–Meier method. Hypothesis testing was conducted on two-sided exploratory 5% significance levels.

**Results:**

373 consecutive pts were enrolled. EP classified 238 pts (63.8%) as low risk and 135 pts (36.2%) as high risk. Median follow-up was 41.6 months. Risk for disease recurrence or death in EPclin high-risk patients was twofold higher in comparison with EPclin low-risk patients (hazard ratio (HR) 2.05 (95% CI 0.85–4.96; *p* = 0.110). Patients with EPclin high risk were at significant higher risk of distant metastases than patients with EPclin low risk (HR 5.18; 95% CI 1.04–25.74; *p* = 0.0443). EPclin high-risk patients who actually underwent adjuvant CTX had a 3-year-DFS of 96.3% (95% CI 92.2–100) in contrast to EPclin high-risk patients without CTX (3-year-DFS: 91.5% (95% CI 82.7–100%); HR 0.32; 95% CI 0.10–1.05; *p* = 0.061).

**Conclusion:**

These first prospective outcome results show that EP, in clinical routine, is a valid clinico-molecular test, to predict DFS and to guide decision of adjuvant CTX use in ER-positive, HER2-negative early breast cancer pts with 0–3 positive lymph nodes. Adjuvant CTX seems to be beneficial for EPclin high-risk patients.

## Introduction

In patients diagnosed with early breast cancer, the lifetime risk for relapse and development of metastases is still high. The main focus of adjuvant treatment is the avoidance of such relapses. In estrogen receptor (ER)-positive disease, the most challenging issue patients and oncologists are faced with is the decision whether neoadjuvant or adjuvant chemotherapy (CTX) should be recommended in addition to endocrine systemic therapy (ET). National and international guidelines recommend adjuvant CTX for patients at high risk for recurrence, while low-risk tumors are to be treated without the use of adjuvant chemotherapy [1, 2]. This implies that accurate risk stratification plays a key role in chemotherapy decision making when treating patients with ER-positive, HER2-negative early breast cancer. Validated prognostic markers are needed to perform such risk stratification. Recognized conventional prognostic markers include tumor size, nodal status, histologic grade, Ki-67, histologic subtype and age. In particular, the current proliferation marker Ki-67 and grading have been shown to be inconsistent within different pathologists [3]. This is one of the reasons why these markers are viewed insufficient to obtain adequate risk stratification in a ER-positive, HER2-negative population [4]. Over the last decade, a variety of new molecular tests based on multigene signatures of the tumor have been developed to overcome this deficit. The 2019 St. Gallen Consensus Conference stated that genomic assays are valuable tools for determining whether adjuvant CTX should be recommended or not [5].

One of these widely used commercially available genomic assays is the EndoPredict^®^ test (Myriad International GmbH, Cologne, Germany). EndoPredict (EP) is a RNA-based 12-gene expression assay which can be carried out on formalin-fixed, paraffin-embedded (FFPE) tumor tissue. It measures the expression of three proliferative and five ER signaling-associated genes, together with four normalization and control genes, by quantitative real-time polymerase chain reaction (qRT-PCR) in decentralized laboratories [6]. By combining the 12-gene molecular score with the clinical risk factors tumor size and nodal status, the EPclin score is generated resulting in low- or high-risk categories. EPclin has been shown to predict early and late recurrences in both postmenopausal patients who received ET and pre- and postmenopausal patients who received ET and CTX as adjuvant treatment for ER-positive/HER2-negative primary breast cancer [7–10]. Moreover, a recent retrospective joint analysis of five prospectively planned trials by Sestak et al. showed that high EPclin scores can predict chemotherapy benefit in women with ER-positive, HER2-negative disease [11]. Because of this level IB evidence, the American Society of Clinical Oncology as well as the European Society of Medical Oncology include EP as a biomarker to guide adjuvant treatment decision in their latest clinical practice guidelines [12, 13]. In a former decision impact study, our group was able to demonstrate that using EPclin as a risk stratification tool in routine clinical practice results in substantial avoidance of adjuvant CTX in endocrine-sensitive, HER2-negative breast cancer [14]. To evaluate the outcome of patients, whose adjuvant CTX decision had been driven by EPclin, we prospectively conducted the present study.

The aim of this study was to collect outcome data of patients with early ER-positive/HER2-negative breast cancer in whom the decision regarding recommendation of adjuvant CTX was based on the EPclin test result. Here we present the first prospective outcome data for EP in routine clinical practice.

## Materials and methods

### Patients and tumour samples

Patients with ER-positive, HER2-negative early breast cancer with 0–3 positive lymph nodes were enrolled at the interdisciplinary breast centre of Klinikum rechts der Isar, Technical University Munich, Germany, between March 2012 and March 2015. Prospective EP testing was carried out on all tumor samples. Demographic, clinical and pathological data drawn from clinical databases and pathological reports were assessed for each patient at baseline. All patients underwent curative surgery. Therapy recommendations were given for all patients during an interdisciplinary tumor board discussing each case individually. Decision for or against chemotherapy was primarily based on the EPclin risk classification, taking individual comorbidity into account. In every case, the recommended and also the performed treatment was documented. Follow-up for each patient was recorded including treatment compliance, local recurrence, distant metastases and survival. Cut-off date of last follow-up was July 31st, 2017.

### EndoPredict analyses

EndoPredict assays (Myriad International GmbH, Cologne, Germany) were performed on FFPE tissue samples of primary breast tumours in the Institute of Pathology at Klinikum rechts der Isar, Technical University Munich, according to the manufacturer’s instructions as described previously [8]. The validated cut-off value of 3.3 for the EPclin score was used for risk discrimination. Patients with an estimated risk of distant recurrence of more or equal to 10% at 10 years were categorized as high risk.

### Statistics

The distribution of quantitative data is described by median (range). Qualitative data are presented by absolute and relative frequencies. Survival analysis is reported using 3-year disease-free survival (DFS) as the primary time to event endpoint. A DFS-event was defined as any recurrence (local, locoregional or distant) or death (with or without recurrence). Distant metastasis-free survival (DMFS) was defined as survival without any distant recurrence. Censored time-to-event outcomes were analyzed by Cox proportional hazards models. Additional estimates of the event-free survival were given by the Kaplan–Meier method. Hypothesis testing was conducted on two-sided exploratory 5% significance levels. Analyses were performed with R 3.6.1 (The R Foundation for Statistical Computing, Vienna, Austria).

## Results

### Study population

A total of 373 patients were enrolled. The median age of the patients was 59.9 (range 29.1–88.9) years. Detailed tumor characteristics are listed in Table 1. Within the 90 patients, whose nodal status was pN1(1–3), 22 (24%) were tested as EPclin low and 68 (76%) as EPclin high.

**Table 1:** Tumor characteristics

Characteristic	*n* (%)	EPclin low, *n* (%)	EPclin high, *n* (%)
Tumor size			
pT1a	20 (5.4)	18 (4.8)	2 (0.6)
pT1b	64 (17.2)	55 (14.7)	9 (2.5)
pT1c	146 (39.1)	106 (28.4)	40 (10.7)
pT2	131 (35.1)	54 (14.5)	77 (20.6)
pT3	12 (3.2)	5 (1.3)	7 (1.9)
Tumor subtype			
Ductal	264 (70.8)	161 (43.2)	103 (27.6)
Lobular	70 (18.8)	48 (12.9)	22 (5.9)
Ductulo–lobular	19 (5.1)	15 (4.0)	4 (1.1)
Others	20 (5.3)	14 (3.8)	6 (1.6)
Grading			
G1	70 (18.8)	60 (16.1)	10 (2.7)
G2	240 (64.3)	161 (43.2)	79 (21.1)
G3	63 (16.9)	17 (4.6)	46 (12.3)
Nodal status			
pN0	283 (75.9)	216 (57.9)	67 (18.0)
pN + (1–3)	90 (24.1)	22 (5.9)	68 (18.2)
PR status			
≥ 20%	301 (80.7)	209 (56.0)	92 (24.7)
< 20%	72 (19.3)	29 (7.8)	43 (11.5)

### EndoPredict test results and recommendation of adjuvant chemotherapy

**Fig. 1:**
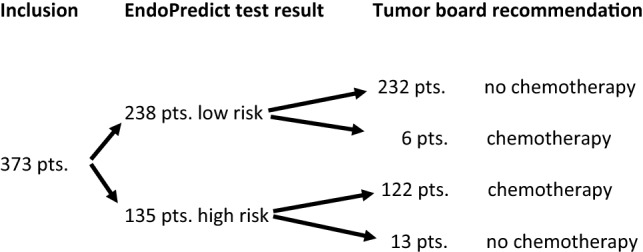
Flowchart demonstrating EndoPredict test results and final tumor board recommendations

The EndoPredict test results and the tumor board recommendations are shown in Fig. 1. The EPclin test was carried out on all 373 tumor samples. The test result allocated 238 patients (63.8%) in the low-risk group and 135 patients (36.2%) in the high-risk group. Six of the 238 EPclin low-risk patients (2.5%) were recommended to undergo CTX even though EPclin was low risk. Reasons for that were young age, multicentric tumor and contralateral breast cancer. 13 of the 135 EPclin high-risk patients (9.6%) were recommended not to undergo CTX even though EPclin was high risk. In these cases, risk–benefit ratio of CTX was not seen as favorable because of patients’ individual risk factors like age or comorbidities.

### Therapy recommendation and compliance

**Fig. 2:**
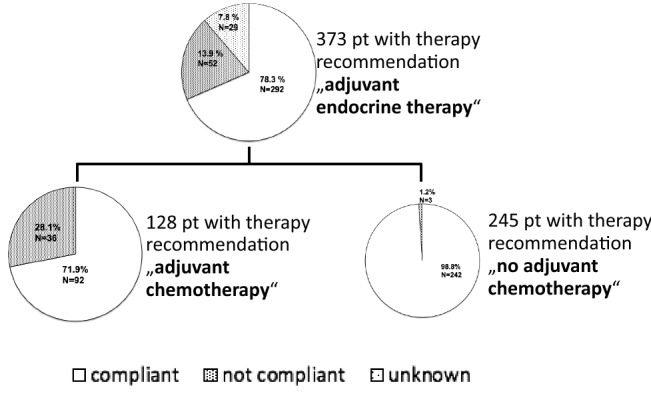
Systemic therapy recommendations given by the interdisciplinary tumor board and patients’ corresponding compliance rates

Figure 2 shows the systemic therapy recommendations given by the interdisciplinary tumor board and the corresponding compliance rates obtained from follow-up data. Adjuvant ET for at least 5 years was recommended in all cases (373 patients). At the time of the last follow-up, 292 (78.3%) patients were compliant and still taking their ET. 52 patients (13.9%) declined ET. For 29 patients (7.8%), information concerning compliance to ET could not be obtained. Adjuvant CTX was recommended in 128 of 373 cases (34.3%). 92 (71.9%) of these patients were compliant and received standard of care CTX whereas 36 patients (28.1%) refused the recommended adjuvant CTX. No adjuvant CTX was recommended in 245 of the 373 cases (65.7%). 3 patients (1.2%) out of these 245 patients underwent CTX without tumor board recommendation. Adjuvant radiotherapy (RTX) was advised in 282 patients (75.6%). 26 (9.2%) of these patients were not compliant, refusing RTX.

### Outcome

**Fig. 3:**
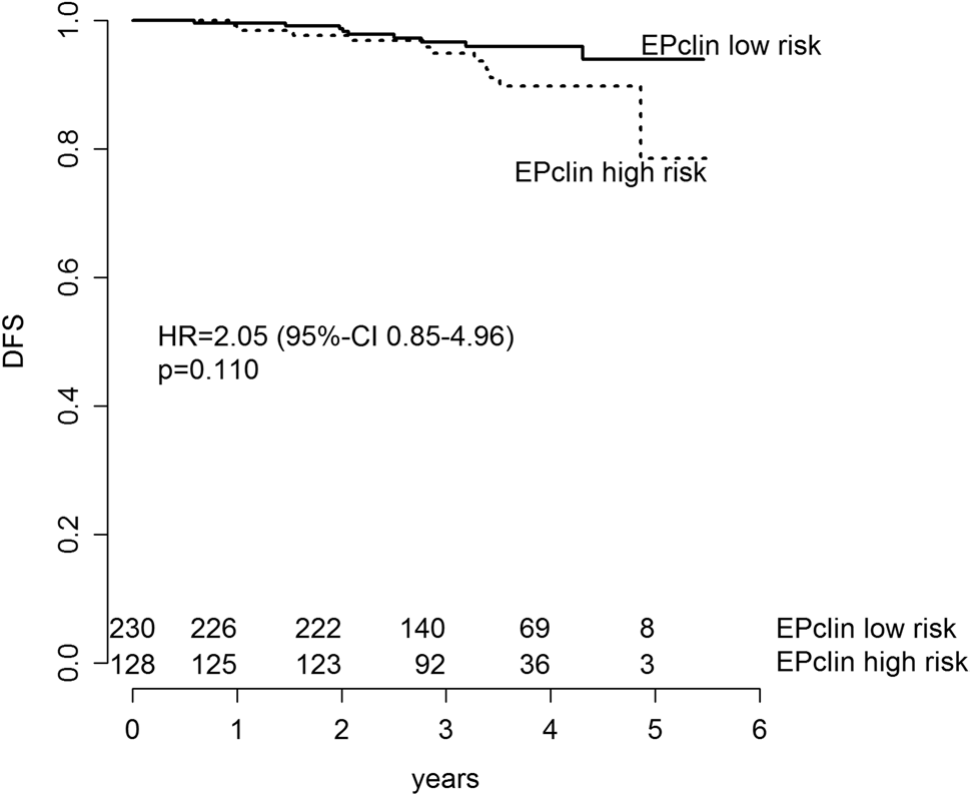
DFS in EPclin low-risk patients and EPclin high-risk patients. Kaplan–Meier estimates of DFS are shown by EPclin risk classification

**Fig. 4:**
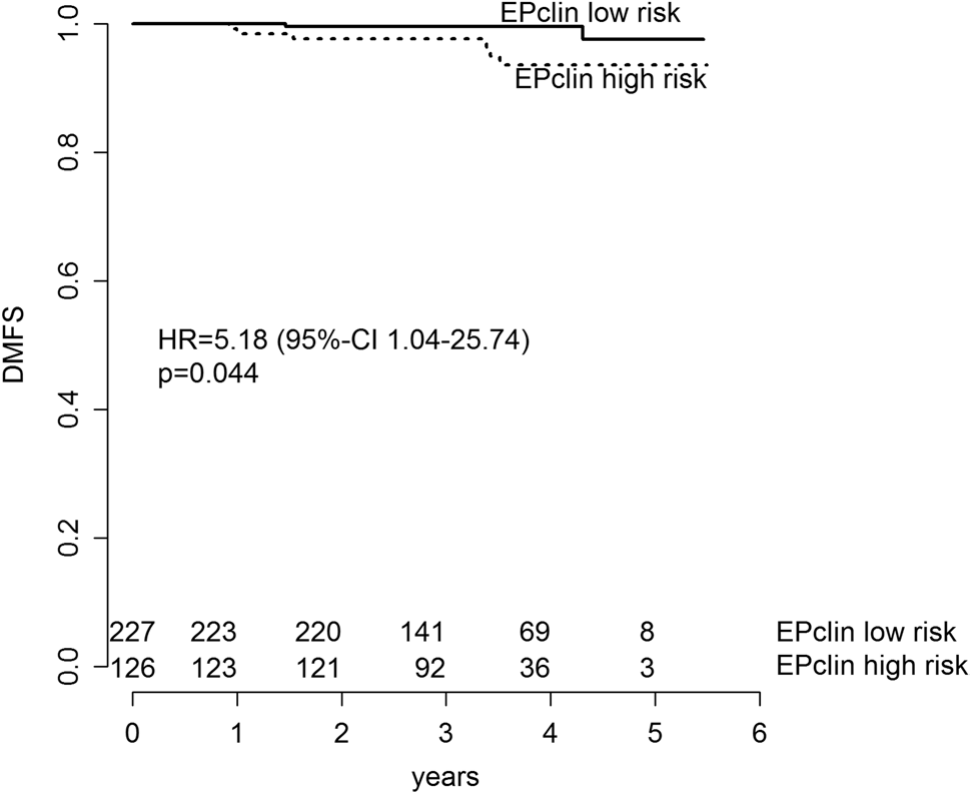
DMFS in EPclin low-risk patients and EPclin high-risk patients. Kaplan–Meier estimates of DMFS are shown by EPclin risk classification

After 41.6 (range 1.6–65.9) months of median follow-up, 22 disease-free survival (DFS) events (11 deaths, 8 distant recurrences, 3 locoregional recurrences) had been observed. Three-year DFS in the whole study cohort was 96% (95% CI 93.8–98.2%). Three-year disease-free survival (DFS) and distant metastasis-free survival (DMFS) in the EPclin low-risk group was 96.6% (95% CI 94.2–99.1%) and 99.6% (95% CI 98.7–100%) versus 94.9% (95% CI 90.9–99.0%) and 97.6% (95% CI 95.0–100%) in the EPclin high-risk group. With a hazard ratio (HR) of 2.05 (95% CI 0.85–4.96; *p* = 0.110), risk for disease recurrence or death in EPclin high-risk patients was twofold higher than in EPclin low-risk patients (Fig. 3). Moreover, patients with EPclin high risk were at significantly higher risk of experiencing distant relapse than patients with EPclin low risk (HR 5.18; 95% CI 1.04–25.74; *p* = 0.0443) (Fig. 4).

**Fig. 5:**
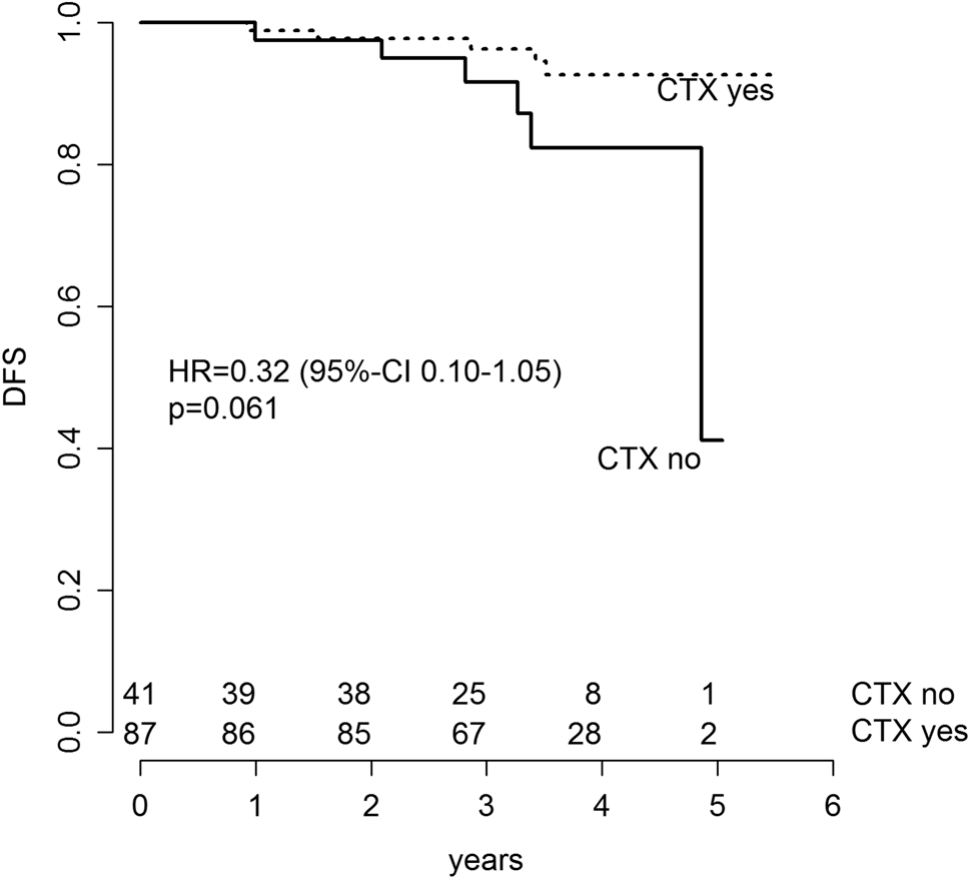
DFS of EPclin high-risk patients, who actually received the recommended adjuvant CTX and DFS of EPclin high-risk patients, who were not compliant with the recommended adjuvant CTX. Kaplan–Meier estimates of DFS of EPclin high-risk patients are shown by CTX compliance

Figure 5 exclusively focuses on DFS of EPclin high-risk patients. EPclin high-risk patients who underwent adjuvant CTX had a 3-year-DFS of 96.3% (95% CI 92.2–100%) and were at lower risk for death or recurrence than those EPclin high-risk patients who did not receive CTX (3-year-DFS: 91.5% (95% CI 82.7–100%); HR 0.32; 95% CI 0.10–1.05; *p* = 0.061). EPclin high-risk patients who received standard adjuvant CTX experienced a 68% reduction in relapse compared to those patients who decided not to undergo the recommended CTX.

## Discussion

In this study, prospective outcome data from a cohort of 373 patients whose adjuvant therapy decision was based on risk stratification with the multigene test EndoPredict are presented. To our knowledge, these are the first prospective clinical outcome data for the multigene test EndoPredict.

The most important finding is the fivefold increased risk for distant metastases in EPclin high-risk patients versus EPclin low-risk patients (HR 5.18, *p* = 0,04). This is in line with formerly published data from retrospective analyses using EP as a prognostic biomarker in ER-positive, HER2-negative patient cohorts; EP could be shown to be an independent predictor of distant recurrence based on retrospective analyses of prospectively collected clinical data from the ABCSG6&8 and TransATAC [10, 15]. The hazard ratios for distant recurrence in EPclin high-risk versus EPclin low-risk patients in these studies were reported as 4.77 and 5.99, respectively, and thus lie in the range of our findings.

In our study, EPclin allocated almost two thirds of the patients into the low-risk group while one-third was classified as being at high-risk for distant recurrence. This is consistent with other previously published real-world data: A French prospective multicenter trial on decision impact included 201 patients, of which 67% were classified as low risk by EPclin [16]. A smaller German study of 82 cases reports an EPclin low-risk rate of 68% [17]. This underlines the robustness of the EndoPredict test in clinical routine.

In this study, we also collected data on patients’ compliance with the adjuvant therapy recommendation given during individual case discussion in the interdisciplinary tumour board. After a median follow-up of 3.5 years, 14% of patients in our study were noncompliant regarding the recommended ET. This compliance rate corresponds with data, where non-adherence to adjuvant ET is reported as being very common, with estimates of up to 50% of patients not successfully completing a 5-year course of treatment [18–20]. Retrospective studies based on databases and registers have shown adherence to adjuvant endocrine therapy of approximately 60–82% and 46–73% after 3 and 5 years, respectively [21, 22].

Since adjuvant CTX leads to significant outcome improvement in endocrine-sensitive breast cancer [23], we analysed whether patients with recommendation for adjuvant CTX actually did receive this treatment. Of the 128 patients who were recommended to undergo CTX in addition to ET, 36 patients did not receive adjuvant CTX, accounting for a CTX non-compliance rate of 28%. Even though this rate of chemotherapy non-compliance at first glance might deem to be surprisingly high, it complies with existing evidence from the German prospective multi-center study BRENDA II. Here 28% of the patients who were allocated to an intermediate-risk group according to conventional clinicopathological prognostic factors, and were recommended to undergo adjuvant CTX by the interdisciplinary tumor board, did, in fact, not adhere to this recommendation and thus did not receive adjuvant CTX [24].

To further elucidate the potential benefit of adjuvant CTX in EPclin high-risk patients of our study cohort, we compared DFS of patients who did, versus did not, receive the recommended adjuvant CTX. We found a remarkable 68% reduction in the risk for death or relapse after 3 years by administration of adjuvant chemotherapy (HR 0.32; 95% CI 0.10–1.05; *p* = 0.06). These data can be interpreted as a prospective confirmation of a study latterly published by Sestak et al.: within a comparative analysis of 3746 patients, they showed that the rate of increase in risk for distant recurrence in patients with high EPclin score can be reduced by administration of adjuvant CTX in addition to ET [11]. Thus, both our and Sestak et al.’s study give evidence that EP indeed can be used to identify patients who might benefit from chemotherapy in ER-positive, HER2-negative breast cancer. Conversely, EP allows the identification of a group of patients who can safely forgo the use of adjuvant CTX; in our study, patients with EPclin low risk experienced a favourable 3-year DMFS-rate of 99.6% (95% CI 98.7–100%). It seems to be unlikely that longer follow-up will result in a substantial change of this positive outcome without adjuvant chemotherapy since the main efficacy of adjuvant chemotherapy is known to evolve during the first 3–5 years after diagnosis [25]. Recently published data on long-term prediction of distant recurrence using EP support this: Filipits et al. reevaluated the combined ABCSG-6/8 cohorts with longer follow-up and reported a 15-year distant recurrence-free rate of 93.4% for EPclin low-risk patients [15].

There are certain limitations to this study. They include the relatively short follow-up period, the moderate sample size and the monocentric, non-randomized design. The discussed early results need to be confirmed with longer follow-up, which will be available in the near future. The results then might also support existing evidence from published retrospective studies showing that EP as a second-generation multigene test is able to extend its prognostic value to prediction of late recurrences [7, 15]. Of note, the international, multicenter study “RESCUE” (“Reaching for evidence based chemotherapy use in endocrine-sensitive breast cancer”, NCT 03503799) has started recruitment in Germany [26]. The study documents distant metastasis-free survival, disease-free survival and overall survival events in patients whose tumors had been tested with EP, and will allow further confirmation of our results.

## Conclusion

In summary, this study for the first time provides prospectively collected real-world data on relapse-free survival of patients, whose adjuvant treatment recommendation was based on using the EndoPredict assay as a prognostic biomarker. Data show that EP in clinical routine is a valid clinico-molecular marker to predict DFS and that adjuvant CTX is beneficial for EPclin high-risk patients. EP can be safely used to guide decision regarding adjuvant CTX in ER-positive, HER2-negative early breast cancer patients with 0–3 positive lymph nodes.

## Data Availability

All relevant data are within the manuscript. Data cannot be made publicly available for ethical or legal reasons; public availability would compromise patient confidentiality or participant privacy.
